# Supplementary material for: Biodiversity resilience in a tropical rainforest
Source: Nature. 2026 Apr 8;652(8112):1232–9. doi: 10.1038/s41586-026-10365-2 (PMC13128449; doi:10.1038/s41586-026-10365-2)
Supplement: Supplementary file 1 — Supplementary Methods, Notes 1 and 2 and References. [file 41586_2026_10365_MOESM1_ESM.pdf]

nature portfolio

https://doi.org/10.1038/s41586-026-10365-2

Supplementary information

Biodiversity resilience in a tropical rainforest

In the format provided by the authors and unedited

Supplementary Information

Supplementary Methods

Survey methods for each taxon

Bacteria: Soil samples were collected with the help of a soil penetrometer at 10 cm and 50 cm depth at 32 plots. At each plot 4 samples were taken for both depths. Upon collection, the respective samples were stored in 2mL microcentrifuge tubes containing 1mL of DNA Shield Buffer (Zymo Research) and were stored in the fridge at 4°C until further processing. The DNA extraction was conducted with the ZymoBIOMICSTM 96 DNA Kit and followed the manufacturer's protocol with these modifications: 400 µl of the sample and 400 µl of DNA shield were added to ZR BashingBead™ Lysis Tubes. Mechanical lysis was performed via bead beating in a vortexer at 2000 rpm for three 15-minute intervals, with 2-minute breaks between each. 16S amplicon sequencing was performed according to the protocol of Kozich et al. (2013)[1], including PCR amplification, normalization using SequalPrep Normalization Plates (Invitrogen) and cleanup with AmPure XP (Beckman Coulter). Libraries were quality checked using a Quibit fluorometer and dsDNA HS kit (Thermo Fisher) and Bioanalyzer High Sensitivity DNA Chip (Agilent Technologies). Sequencing was performed on an in house Illumina MiSeq using 2x250 cyc v2 chips. Bioinformatic cleanup, processing including denoising and ASV generation, and taxonomic classification was performed according to the https://github.com/chiras/metabarcoding_pipeline workflow utilizing VSEARCH as a main tool. Further cleanup steps and transformation to relative read abundances per sample were performed with phyloseq in R also according to the script above. Samples with less than 15.000 quality-controlled reads were removed from the analyses. If multiple samples for both investigated samples were available after quality control, the sample with the highest throughput was selected for further analysis.

Leaf-litter arthropods: In spring 2022 we sampled 30 study plots, covering five forest types (8 old-growth forest plots, 3 active cacao plantations, 3 active pastures, 8 secondary forests recovering from use as cacao plantation and 8 secondary forests recovering from use as pasture). The sampling consisted in taking 1 m2 of leaf litter in each study site and then sieve it with a 1 cm mesh to upconcentrate the arthropods. The resulting subsample was then brought to the lab and Berlese-extracted for 60 hours. The arthropods were collected and stored in 96% EtOH until identification. The arthropods were sorted first into orders and then then into

morpho species. Diptera, larvae and Formicidae were removed from this dataset which comprises a total of 4067 individuals classified in 377 morpho-species.

Saproxylic beetles: All 62 study plots were sampled. To assess the potential specialization of saproxylic insects towards specific host trees, we quantified dead wood × insect networks using logs from five phylogenetically distinct local tree species in a standardized way along a forest recovery gradient. Logs (approximately 6-12 cm in diameter and 50 cm in length) were cut from branches or stems of young healthy trees with no visible signs of insect or fungal activity. Specifically, *Trema micrantha* (Sapanillo) and *Theobroma cacao* (Cacao) were selected as fast-decomposing species; *Inga* spp. (Guaba) as a medium-decomposing species; and *Triplaris cumingiana* (Fernán Sánchez) and *Hieronyma chocoensis* (Mascarey) as slow-decomposing species based on local knowledge. The logs were placed on the ground in each plot for six months, following a similar protocol to a global experiment[2].
We exposed a total of 310 logs in the plots. When removing the logs from the field, we first collected surface-active ants and termites using the logs as nesting space. Before placing the logs in the emergence chambers, a wooden disc was cut from each end of the log for subsequent characterization of fungal communities using metabarcoding. The emergence chambers consisted of net tubes made from mesh cloth used for insect nets. All emerging insects were collected in tubes hanging at the lower end of the emergence chambers. Beetle species were initially identified as morphospecies within the subfamily, and one individual per sample and morphospecies was selected for subsequent barcoding.

Dung beetles: We sampled all 62 study plots. The first round of sampling was conducted from September to November 2021, and the second round from March to May 2022. Within each 50 m x 50 m study site, four pitfall traps were set 50 m apart to avoid trap interference. The traps were one-quarter filled with 70% Ethanol, and two leaves were placed over the top as rain protection. Each trap had a different bait: cow dung, rotten cow muscle, rotten millipede, or fermented fruit (banana). The traps were collected after 48 hours, emptied into small containers, and transported back to the lab. The dung beetles were separated from the other arthropods in the lab and stored in pure Ethanol. Diego Marin-Armijos identified the beetles as precisely as possible. We captured 844 dung beetles in the six months of fieldwork. Two of the 23 species are undescribed and remain morphospecies.

Nocturnal insects (metabarcodes): We sampled nocturnal insects with an autonomous light trap for one night per plot. These traps were equipped with an LED light optimized for insect sampling powered by a Power bank (LepiLED Mini, Brehm, Jena Germany[3]). For 8 hours after dusk, insects were collected in a jar mounted under a funnel and killed by chloroform. We removed large-bodied Lepidoptera from the Saturnidae and Sphingidae families (moths) for taxonomic identification. The remaining insect bulk was frozen and transferred to 96% alcohol. The samples were then passed through an 8-mm sieve, thereby separating larger and smaller insects, opening the avenue for using read numbers for abundance estimates. This was done to increase the likelihood of detecting small-bodied and rare species in samples, as individuals with a larger biomass provide disproportionate amounts of DNA and can therefore be over-represented when metabarcoding bulk samples. The CO1-5P (mitochondrial cytochrome oxidase 1) target region was sequenced for collected bulk samples. These COI sequences were used to attribute Barcode Index Numbers (BINs), which are clusters of barcode sequences that can be used as a proxy taxonomic unit. The BIN clusters match the actual taxonomically identified species at different levels (90–99% COI genetic similarity), depending on the taxa, thus allowing comparisons with studies based on morphological determination. The reference library was restricted to countries from Central and Southern America (for more details see Müller et al. 2023[4]). Sequencing errors were removed according to Chiu et al. (2016). [5]

Moths: The two focal groups of moths (Sphingidae and Erebidae: Arctiinae) were collected at the 62 plots belonging to the unit. Thirty-two plots were sampled between March and May 2022 and the remaining 30 between October and December 2022. A similar proportion of plot types (actively used cacao plots, actively used pasture plots, secondary forest plots with legacy of cacao use, secondary forest plots with legacy of pasture use, and old-growth forest plots) were sampled in the two seasons. Moths were captured with a white vane trap equipped with a LepiLED Maxi Switch light[6] set to emit a mixed light (UV and visible white light). The vanes were connected to a collection and killing chamber, where chloroform fumes were used as a dry and fast-killing agent. The trap was protected from rain by a wide plastic roof. At all plots we set one trap at the understory (ca. 1.5 m above ground), and at older plots (secondary forest plots with legacy of cacao use, secondary forest plots with legacy of pasture use with more than 15 years of regeneration and that had a clear vertical structure and at least one tree taller than 15 m, and all old-growth forest plots), we also set an additional canopy trap ranging from 15 – 35 m above ground using a pulley system set with the aid of a bow (N = 27). Using a digital timer, the lights were set to turn on at 1800 and remained working for 12 hours. Lights were

connected to a power bank. The following morning, moths were collected and filtered according to our focal groups. Individuals were identified to the (morpho)-species level based on a personal catalog curated by Dr. Gunnar Brehm (Phyletisches Museum Jena, Germany). We recorded a total of 11902, from which 11271 could be identified to (morpho)-species level (279 species recorded).

Bees: Bees were collected using a variety of methods. Between March and May 2022 and between October and December 2022, diurnal bees were collected using a modified vane trap similar to commercially available models[7], adapted to with a yellow and a blue vane attached transversally to attract a wider range of bees. The collection and killing chamber, roof, canopy setting and number of plots sampled per season was the same as described above for moths. Traps were always set in the morning and emptied 24 hours later. Additionally, in 2022, nocturnal *Megalopta* (Halictidae) and *Ptiloglossa* (Colletidae) bees were often collected by the moth traps described above.

In 2023, vane traps were set again similarly to 2022 and in the same plot setting, this time with added honey baits at the bottom of the vanes to increase capture rates. Additionally, fragrance traps, targeted at male orchid bees (Apidae: Euglossini), were set simultaneously to the vane traps. These traps were modified 2L PET bottles with two entrance holes guided by plastic funnels. We used four traps per plot (and per stratum when the canopy was sampled, see "Moths" section above), each with one of the following scents known to attract different species of orchid bees[8]: 1,8-cineole, Eugenol, Methyl Salicylate, and Skatol. A piece of cotton imbibed in common household insecticide was used as a fast-killing agent. Lastly, sessions of hand-netting were carried out in the plots after traps were set. Four hours of free walking were carried out within the 50x50 m area of each plot, in which all bees were captured on sight and stored individually for sorting and identification. Bees were identified through specialized keys for each group (e.g., refs. 9-11).

Ants: Ants were collected on all 62 plots in February-April 2022 by hand sampling of the ground and trees and with the use of Winkler traps as described by Hoenle et al. (2022)[12]. In the same sampling period, five naturally occurring dead wood pieces per plot were opened and insects nesting in the dead wood were collected. Additionally, ants were collected in an experimental approach on 62 plots with wood from five different tree species. In August-September 2022 one piece of wood with a diameter of 7-10 cm and a length of 50 cm originating from the tree species *Trema micrantha* (Sapanillo), *Theobroma cacao* (Cacao),

Inga sp. (Guaba), *Triplaris cumingiana* (Fernán Sánchez) and *Hieronyma chocoensis* (Mascarey) was placed on each plot. After 6 months in February-April 2023 the wood was retrieved from the plot and reared in emergence chambers. These emergence chambers consisted of mesh tubes made of fabric used for insect nets and a falcon tube filled with ethanol. Insects emerging from the wood and falling into the ethanol were collected for the following three months. Ant species were identified with a genetical approach using the COI barcoding gene in combination with the examination of morphological traits.

Frogs: We sampled 38 plots. Each plot was visited six times over three rounds, with each round including both day and night sampling sessions. The first round took place from March to June 2022, the second from July to October 2022, and the third from March to June 2023. In each plot, we performed systematic searches for two person-hours, visually scanning for frogs from the leaf/litter up to vegetation approximately 1.2 meters in height. While our focus was on leaf-litter frogs, we recorded all frog species encountered. Each frog was caught and temporarily placed in an individual plastic bag until the end of the sampling. Frogs were then identified using BioWeb[13]. To minimize temporal effects on the results, plots from different habitat types were sampled alternately.

Ground birds and ground mammals: Two different datasets, sampling both ground birds and ground mammals using camera traps, were combined for this study and then split into ground birds and ground mammals.

Dataset 1: Frugivorous birds and mammals on the forest floor were recorded across the 62 study plots using camera traps. In each plot, fruits of fruiting plant species growing at the plot were deployed in front of the camera traps and continuously recorded for six days. Cameras were positioned at each of the four corners of each plot. Sampling was carried out from 2022 to 2023 during both rainy (March-June) and dry (September-December) seasons. We recorded all events where ground-dwelling frugivores consumed fruits. Birds were identified following Restall & Freile (2018)[14] and mammals following Tirira (2017)[15]. The total interaction frequency was used as a proxy for animal abundance. In total, we recorded 13 bird species interacting 70 times and 9 mammal species interacting 137 times with fruits on the forest floor across all plots. All observed interactions, including those involving cultivated plants, are included in this study.

Dataset 2: Ground birds and mammals were captured by camera traps as described in Grella et al. (in press)[16]. In short, sampling took place in the dry season from October - November in

the years 2021 and 2022. One camera (Reconyx Hyperfire) was installed on each plot over the course of three weeks and retrieved in the same order resulting in three weeks of operating time for each camera. Cameras were installed on trees at 30-80 cm height facing open areas or animal trails. 65 plots (3 plots were part of an older study design) were sampled, but due to malfunctions of some cameras or vegetation blocking the vision 8 plots were excluded, resulting in 57 successfully sampled plots. Cameras were deployed to take three images per trigger event with 1 second of delay between triggers. Species were identified using the management software TRAPPER[17] and literature about local mammal and bird fauna[18–20]. For assessing the number of species occurrences, individual birds and mammals were counted. As individuals of most species cannot be distinguished, we counted individuals of the same species as a new occurrence after a minimum of 5 minutes from the last image or sequence of images[21,22].

Birds (sound data): We deployed one Bioacoustic Recorder (BAR-LT, Frontier Labs, Meanjin, Australia) with one omnidirectional microphone facing down at a height of ca. 1.70 m above ground, at each plot during November for several weeks. The recorders were programmed to record 2 minutes every 15 minutes throughout the day for two weeks (Julian day 299-314 in 2021) concurrently, with a sampling rate of 44.1 kHz. Two experts identified all birds from 2-minute files recorded at 06:00h, 06:15h, 06:30h, 06:45h, 07:00h, 07:15h, 12:00h, 12:15h, 16:00h, 16:15h, 17:00h, 17:15h, 18:00h, 18:15h from two days without heavy rain, covering the high activity phases of birds around dusk and dawn, as well as few minutes during the day to cover flock activities. For more details see Müller et al 2023[4], Falconi et al 2024[23].

Frugivorous birds: Frugivorous birds were recorded across all 62 study plots through direct observations of the upper forest layers (midstory and canopy) using binoculars. Each plot was observed for 5 hours starting at sunrise over three consecutive days, resulting in 15 hours of observations per plot. Sampling was carried out from 2022 to 2023, during both rainy (March-June) and dry (September-December) seasons. Bird species were identified following Restall & Freile (2018)[14]. We recorded all events where birds consumed fruits. The total interaction frequency of each bird species was used as a proxy for bird abundance. In total, we recorded 68 bird species interacting 1597 times with fruits across all plots. This study contains all bird-plant interactions, including those involving cultivated plants.

Bats: Bat sampling was conducted at all 62 sites in four field campaigns, from March 2022 to December 2023, during the dry and rainy seasons. Mist nets have proven to be an effective method for capturing phyllostomid bats[24], which are our study group. At each site, six mist nets (6m x 2.5m) were set at ground level and captures were made on three consecutive nights, for 186 sampling nights. They were open from 18h30 to 24h00 (5.5 hours) and were checked approximately every 30 minutes. The total sampling effort was 6138 hours/net. Guidelines such as Sikes et al. (2016)[25] and Erazo et al. (2022)[26] were followed for handling and manipulating bats. For the registration of the captured individuals, general morphometric measurements were taken, as well as data on sex, age, and reproductive status. Identification and taxonomic classification were carried out using guides, keys, and updated species lists (e.g., Díaz et al. 2021[27]; Tirira et al. 2017[15]; Tirira et al. 2024[20]; Simmons and Cirranello 2024[28]). To identify recaptures, a temporary mark was applied to each individual, with numbered punches placed in tweezers to make small holes in the membrane of the extended right-wing[25,29,30]. As a reference collection, 67 individuals were deposited in the Mammal Collection of the Zoology Museum of the Pontificia Universidad Católica del Ecuador (QCAZ-M), registered under the Contrato Marco MAAE-DBI-CM-2021-0165, issued by the Ministry of Environment of Ecuador.

Seedlings: We monitored tree seedling recruitment on four 1-m2 subplots on 39 plots. We defined seedlings as all young plants (including palm trees), which grow from a seed or resprout from a fallen branch and have a root-collar diameter (RCD) between 2 mm and 10 mm. All tree seedlings were tagged and identified to the lowest taxonomic level possible (species or morpho-species level). For every seedling we measured its height, RCD, and number of leaves as well as monitored its survival over 31 months with repeated measurements every four months. For this study, we used the data of the seedling communities gathered in the fourth monitoring (March to May 2023) to calculate taxonomic diversity and composition per plot.

Trees: In each of the 62 study plots, all trees (N =6426), palms (N = 1108) and lianas (N = 8) with ≥ 25 cm of circumference at 1.3 m above the ground (≥ 7.95 cm diameter at breast height, DBH) were labeled and identified in the 50 x 50 m plot area. Within active plots, which were not fenced, we tried to cover the same area as in the 50 x 50 plots (250 m^2) and also tried to include remnant wild trees if these were not too far away from the center of the plot. The tree survey was conducted February 2022 to July 2023. A botanical collection and silica samples for further genetic and chemical analyses were obtained from each tree species. Tree

identification was performed at the Herbario Nacional del Ecuador–INABIO using the collections deposited there as references. From a total 7968 stems of 7542 cultivated and non-cultivated tree individuals (some with several stems), we identified a total of 539 tree species and morphospecies. In total, 7408 tree individuals were identified to species and morphospecies level which is more than 98% of the 7542 trees surveyed. Only 134 tree individuals have not been identified because they did not present leaves during the survey or additional identification work at the herbarium is required. For this study, we use all trees including cultivated ones (798 stems of 678 cultivated trees of 11 species, including cacao, coffee, coconut, and lemon trees common in the area). An alternative meaningful subset, focusing on wild trees and excluding cultivated ones, is reported and analyzed elsewhere (see Escobar et al. 2024[31]). For further details, see Escobar et al. 2024[31]

Supplementary Note 1

Example calculation for resistance, return rates and recovery time of species composition for bees

For each investigated plot we calculated the pairwise Bray-Curtis similarity to each of 17 old-growth forest plots. Thereby, we obtain 17 values and then calculate the mean value of these values (also for each old-growth forest compared to the 16 other old-growth forest plots). Each data point in Fig. 3 and Extended Data Figs. 1 and 2 corresponds to such a mean value of 17 (or 16) comparisons. The exact values can be found in Supplementary Table 1. We fitted a negative exponential function, similar to Poorter et al. (2021)[32], to this data, as given by the equation

$$\psi(t) = \psi_0 + (\psi_{Ref} - \psi_0)(1 - e^{-\lambda t}), \qquad (1)$$

where $\psi(t)$ is the value of the respective index at time t, ψ_0 and ψ_{Ref} are the median of the respective index for the active and old-growth plots, respectively, and λ is the return rate to the old-growth level, termed "intrinsic recovery rate" in ref. 32. This function assumes an asymptotic approach to the old-growth forest level over time, starting from the level of the active agriculture plots. The return rate λ is the parameter of the function that is optimized during the fitting procedure.

To calculate the recovery time, which we define as the time when the ecological metric (in this example the Bray-Curtis similarity), has reached 90% of the old-growth forest level, we solve Eq. 1 for the case $\psi(t) = 0.9 \cdot \psi_{Ref}$ and obtain

$$T_{rec} = \frac{-ln(0.1 \cdot \psi_{Ref}/|\psi_{Ref} - \psi_0|)}{\lambda} \qquad (2)$$

Inserting the example values for bees into Eq. 2 ($\lambda = 0.0658$, $\psi_0 = 0.1825$, $\psi_\infty = 0.4147$), we obtain:

$$T_{rec} = \frac{-ln(0.1 \cdot 0.4147/|0.4147 - 0.1825|)}{0.0658} = 26.18\, years$$

Resistance and resilience are calculated according to the following equations:

$$Resistance = \exp\left(-\left|ln\left(\frac{\psi_0}{\psi_{Ref}}\right)\right|\right) \qquad (3)$$

$$Return\, rate = \lambda \qquad (4)$$

where ψ_0 in this example refers to the median value of the species composition similarity of all active agriculture plots and ψ_{Ref} to the median value of the species composition similarity of all old-growth forest plots. In this example, with $\psi_0 = 0.1825$, $\psi_{Ref} = 0.4147$ and $\lambda = 0.0658$, we obtain:

$$Resistance = \exp\left(-\left|ln\left(\frac{0.1825}{0.4147}\right)\right|\right) = 0.44$$

$$Return\ rate = \lambda = 0.0658$$

The results presented here differ slightly from the results reported in Supplementary Data 1 due to the rounding of values in this example which was done for simplification.

Supplementary Note 2

Justification of treatment of the frugivorous bird, moth and saproxylic beetle dataset

When calculating recovery times of the abundance of frugivorous birds for cacao and pasture we obtained values of $1.66 \cdot 10^{22}$ years and $1.54 \cdot 10^{21}$ years. For the recovery time of the species diversity (Shannon diversity) of moths in cacao we obtained a value of $8.3 \cdot 10^{13}$ years and for the recovery time of the species composition (Bray-Curtis distance) of saproxylic beetles in cacao we obtained a value of $1.8 \cdot 10^{10}$ years. While these high values could indicate a case of arrested recovery, we manually assessed the recovery trajectory (see Extended Data Fig. 7) of these cases and decided to set these two recovery times to a value of 0 years in Figure 2 (marked with an asterisk).

We did so, because the data shows indeed no recovery over time. However, the large spread of values, including the old-growth forest plots, do not allow to draw the conclusion that there is an arrested recovery, but rather that there is no difference, within standard deviation, in active agriculture plots, recovering plots and old-growth forest plots. We thus decided to set the recovery time to 0, which is more plausible based on the data.

References

1. Kozich, J. J., Westcott, S. L., Baxter, N. T., Highlander, S. K. & Schloss, P. D. Development of a dual-index sequencing strategy and curation pipeline for analyzing amplicon sequence data on the MiSeq Illumina sequencing platform. *Appl. Environ. Microbiol.* **79**, 5112–5120 (2013).
2. Seibold, S. *et al.* The contribution of insects to global forest deadwood decomposition. *Nature* **597**, 77–81 (2021).
3. Brehm, G. *et al.* Moths are strongly attracted to ultraviolet and blue radiation. *Insect Conserv. Divers.* **14**, 188–198 (2021).
4. Müller, J. *et al.* Soundscapes and deep learning enable tracking biodiversity recovery in tropical forests. *Nat. Commun.* **14**, 6191 (2023).
5. Chiu, C.-H. & Chao, A. Estimating and comparing microbial diversity in the presence of sequencing errors. *PeerJ* **4**, e1634 (2016).
6. Brehm, G. A new LED lamp for the collection of nocturnal Lepidoptera and a spectral comparison of light-trapping lamps. *Nota Lepidopterol.* **40**, 87–108 (2017).
7. Prendergast, K. S., Menz, M. H., Dixon, K. W. & Bateman, P. W. The relative performance of sampling methods for native bees: an empirical test and review of the literature. *Ecosphere* **11**, e03076 (2020).
8. Cancino, A. D. M. & Damon, A. Fragrance analysis of euglossine bee pollinated orchids from Soconusco, south-east Mexico. *Plant Species Biol* **22**, 127–132 (2007).
9. Bonilla-Gómez, M. A. & Nates-Parra, G. Abejas euglosinas de Colombia (Hymenoptera: Apidae) I. Claves ilustradas. *Caldasia* 149–172 (1992).
10. Roubik, D. W. Stingless bees: a guide to Panamanian and Mesoamerican species and their nests (Hymenoptera: Apidae: Meliponinae) *Insects of Panama and Mesoamerica: selected studies*, 495-524 (1992).
11. Santos, L. M. & Melo, G. A. R. Updating the taxonomy of the bee genus Megalopta (Hymenoptera: Apidae, Augochlorini) including revision of the Brazilian species. *J. Nat. Hist.* **49**, 575–674 (2015).
12. Hoenle, P. O. *et al.* Rapid ant community reassembly in a N eotropical forest: Recovery dynamics and land-use legacy. *Ecol. Appl.* **32**, e2559 (2022).
13. Ron, S., Merino-Vitero, A. & Ortiz, A. BioWeb, Anfibios Del Ecuador. (2024).
14. Restall, R. & Freile, J. *Birds of Ecuador*. (Bloomsbury Publishing, 2018).
15. Tirira, D. *A Field Guide to the Mammals of Ecuador: Including the Galapagos Islands and the Ecuadorian Antarctic Zone*. (Asociación Ecuatoriana de Mastozoología, 2017).
16. Grella, N. *et al.* Vertebrate diversity and biomass along a recovery gradient in a lowland tropical forest. *Biotropica* **in Press**,.
17. Bubnicki, J. W., Churski, M. & Kuijper, D. P. Trapper: An open source web-based application to manage camera trapping projects. *Methods Ecol. Evol.* **7**, 1209–1216 (2016).
18. Ridgely, R. S., Greenfield, P. J., Coopmans, P. & Kalil, G. *Aves Del Ecuador: Guía de Campo*. (Fundación de conservación Jocotoco, 2006).
19. Billerman, S. M., Keeney, B. K., Rodewald, P. G. & Schulenberg, T. S. Birds of the World. *Cornell Lab. Ornithol. Ithaca NY USA* (2022).
20. Tirira, D. G. *et al.* Mamíferos del Ecuador: lista oficial actualizada de especies / Mammals of Ecuador: official updated species checklist. Versión 2024.1. (2024).
21. Rovero, F. & Marshall, A. R. Camera trapping photographic rate as an index of density in forest ungulates. *J. Appl. Ecol.* **46**, 1011–1017 (2009).
22. Henrich, M. *et al.* Deer behavior affects density estimates with camera traps, but is outweighed by spatial variability. *Front. Ecol. Evol.* **10**, 881502 (2022).
23. Falconí-López, A. *et al.* Habitat niches of bird species along a recovery gradient in the Chocó tropical forest. *Ecol. Indic.* **166**, 112260 (2024).
24. Kalko, E. K. V. Organisation and diversity of tropical bat communities through space and time. *Zoology* **101**, 281–297 (1998).
25. Sikes, R. S. & Mammalogists, A. C. and U. C. of the A. S. of. 2016 Guidelines of the American Society of Mammalogists for the use of wild mammals in research and education. *J. Mammal.* **97**, 663–688 (2016).
26. Erazo, S. *et al.* Lineamientos éticos y procedimientos para el estudio y manejo de mamíferos silvestres en el ecuador. *Asoc. Ecuat. Mastozool. Minist. Ambiente Agua Transic. Ecológica Ecuad.* (2022).
27. Díaz, M. M., Solari, S., Gregorin, R., Aguirre, L. F. & Barquez, R. M. Clave de identificación de los murciélagos neotropicales. (2021).
28. Simmons, N. B. & Cirranello, A. L. *Bat Species of the World: A Taxonomic and Geographic Database*. (2024).
29. Bonaccorso, F. J. & Smythe, N. Punch-marking bats: an alternative to banding. *J. Mammal.* **53**, 389–390 (1972).
30. Powell, R. A. & Proulx, G. Trapping and marking terrestrial mammals for research: integrating ethics,

performance criteria, techniques, and common sense. *ILAR J.* **44**, 259–276 (2003).
31. Escobar, S. *et al.* Reassembly of a tropical rainforest ecosystem: A new chronosequence in the Ecuadorian Chocó tested with the recovery of tree attributes. 2024.03.21.586145 Preprint at https://doi.org/10.1101/2024.03.21.586145 (2024).
32. Poorter, L. *et al.* Multidimensional tropical forest recovery. *Science* **374**, 1370–1376 (2021).
