# Supplementary material for: Biodiversity resilience in a tropical rainforest
Source: Nature. 2026 Apr 8;652(8112):1232–9. doi: 10.1038/s41586-026-10365-2 (PMC13128449; doi:10.1038/s41586-026-10365-2)
Supplement: Supplementary file 2 — Reporting Summary [file 41586_2026_10365_MOESM2_ESM.pdf]

nature portfolio

Corresponding author(s): Timo Metz & Nico Blüthgen

Last updated by author(s): 12/12/2024

Reporting Summary

Nature Portfolio wishes to improve the reproducibility of the work that we publish. This form provides structure for consistency and transparency in reporting. For further information on Nature Portfolio policies, see our Editorial Policies and the Editorial Policy Checklist.

Statistics

For all statistical analyses, confirm that the following items are present in the figure legend, table legend, main text, or Methods section.

n/a	Confirmed	
☐	☒	The exact sample size (*n*) for each experimental group/condition, given as a discrete number and unit of measurement
☐	☒	A statement on whether measurements were taken from distinct samples or whether the same sample was measured repeatedly
☐	☒	The statistical test(s) used AND whether they are one- or two-sided<br>*Only common tests should be described solely by name; describe more complex techniques in the Methods section.*
☐	☒	A description of all covariates tested
☐	☒	A description of any assumptions or corrections, such as tests of normality and adjustment for multiple comparisons
☐	☒	A full description of the statistical parameters including central tendency (e.g. means) or other basic estimates (e.g. regression coefficient) AND variation (e.g. standard deviation) or associated estimates of uncertainty (e.g. confidence intervals)
☐	☒	For null hypothesis testing, the test statistic (e.g. *F*, *t*, *r*) with confidence intervals, effect sizes, degrees of freedom and *P* value noted<br>*Give P values as exact values whenever suitable.*
☒	☐	For Bayesian analysis, information on the choice of priors and Markov chain Monte Carlo settings
☒	☐	For hierarchical and complex designs, identification of the appropriate level for tests and full reporting of outcomes
☒	☐	Estimates of effect sizes (e.g. Cohen's *d*, Pearson's *r*), indicating how they were calculated

Our web collection on statistics for biologists contains articles on many of the points above.

Software and code

Policy information about availability of computer code

Data collection	No software was used for data collection
Data analysis	Data and code both are shared in a fully reproducible CodeOcean repository (https://doi.org/10.24433/CO.1040081.v1). We used R version v4.3.2 with the packages "ranger v0.16.0" for random forest analysis and iNEXT v3.0.0 and iNEXT.beta3D v1.0.1 for standardization of alpha- and beta-diversity Hill-numbers. Other R packages used are:lme4 v1.1-36, car v.3.1-3, viridis v. 0.6.5, vegan 2.6-8, MuMIn v. 1.48.11, dplyr v2.5.0, doSNOW v1.0.20, progess v1.2.3, visreg v2.7.0, reshape2 v1.4.4, tidyverse v2.0.0. We used Python version 3.11.4 for analysis of data that has been collected in the context of this work and Python version 3.10.9 in a Jupyter notebook on a notebook server of v6.5.2 for analysis of the literature data. Other python-packages: matplotlib v3.7.0, numpy v1.23.5, pandas v1.5.3, uncertainties v3.1.7, Scipy v1.10.0, adjustText v1.2.0. We used the scipy package v1.10.0 in Python for fitting Eq. 2 in the manuscript to the data. All software and packages are listed in detail in the Methods. Methodological details are also provided in the README file.

For manuscripts utilizing custom algorithms or software that are central to the research but not yet described in published literature, software must be made available to editors and reviewers. We strongly encourage code deposition in a community repository (e.g. GitHub). See the Nature Portfolio guidelines for submitting code & software for further information.

Data

Policy information about availability of data

All manuscripts must include a data availability statement. This statement should provide the following information, where applicable:

- Accession codes, unique identifiers, or web links for publicly available datasets
- A description of any restrictions on data availability
- For clinical datasets or third party data, please ensure that the statement adheres to our policy

Data availability:Raw data is available in an online CodeOcean repository (DOI: https://doi.org/10.24433/CO.1040081.v1). Code availability: All code is available in an online CodeOcean repository (DOI: https://doi.org/10.24433/CO.1040081.v1).

Research involving human participants, their data, or biological material

Policy information about studies with human participants or human data. See also policy information about sex, gender (identity/presentation), and sexual orientation and race, ethnicity and racism.

Reporting on sex and gender	N/A
Reporting on race, ethnicity, or other socially relevant groupings	N/A
Population characteristics	N/A
Recruitment	N/A
Ethics oversight	N/A

Note that full information on the approval of the study protocol must also be provided in the manuscript.

Field-specific reporting

Please select the one below that is the best fit for your research. If you are not sure, read the appropriate sections before making your selection.

☐ Life sciences ☐ Behavioural & social sciences ☒ Ecological, evolutionary & environmental sciences

For a reference copy of the document with all sections, see nature.com/documents/nr-reporting-summary-flat.pdf

Ecological, evolutionary & environmental sciences study design

All studies must disclose on these points even when the disclosure is negative.

Study description	The study are of our collaborative Research Unit "Reassembly" is located in the lowland tropical rainforest within the Ecuadorian Chocó, a biodiversity hotspot that is highly threatened by deforestation. The main aim of this research is to understand the mechanisms, resistance and resilience of a naturally recovering rainforest ecosystem following deforestation.<br>The study design is a chronosequence with a total of 62 plots (50x50m) in cacao plantations and pastures, early and late regeneration (0 - 38 year old secondary forests that were previously used as cacao plantation or pasture), and mature old-growth forests for reference. The study area (ca. 200 km2) offers unique opportunities unmatched by any other study at a similar scale: a highly resolved chronosequence of spatially independent plots of variable age with clear land-use history, maintained and made accessible to research by a conservation foundation.
Research sample	We studied an broad spectrum of animal, plant and bacteria taxa representative for different ecological functions in the forest ecosystem, surveyed im all plots: Ants, Bacteria, Bats, Bees, Dung beetles, Frogs, Frugivorous birds, Ground birds, Leaf-litter arthropods, Mammals, Moths, Nocturnal insects, Saproxylic beetles, Tree seedlings, Trees (Total: 10840 species or morphospecies plus 23590 bacteria sequences).
Sampling strategy	The sample size for our analysis of recovery, resistance and resilience was the number of plots of the chronosequence (62 plots in total). Pilot studies on ants and trees (see references) confirmed the feasability of the study design, the lack of spatial autocorrelation for the selected plots as well as the lack of bias by elevation and landscape parameters (see Escobar et al. 2024, cited in the manuscript).
Data collection	Each taxon was sampled with specific state-of-the-art sampling techniques by the authors. Sampling methods include traps, sound recorders, wildlife cameras, mist netting, standadized observations, extraction methods of soil, litter or deadwood. Details are provided in the Methods.
Timing and spatial scale	Data collection methods were predetermined before the onset of the study, and field work was generally conducted over a long time

Timing and spatial scale	within two years untiul allplots have been sampled. All taxa were recorded simultaneously in this time by different authors. All worked wirthing the same set of plots to allow direct comparability.
Data exclusions	No data were excluded.
Reproducibility	The data in this study are observational and contain no experimental manipulation (except different forms of land use history, namely pasture and cacao plantation that were considered in the analysis). Methods are completely reproducible based on the detailed description in the paper, and can be comparted to our open data.
Randomization	The study design (location of 62 plots) was defined and established prior to the collection of data. Potential spatial biases were controlled for (i.e. variation in elevation and landscape features) and are decribed and analysed in detail in our site description paper (Escobar et al. 2024, cited in the manuscript). Each taxon sampled had a responsible expert principal investigator and a PhD researcher familiar with this taxon; all of them are included as authors.
Blinding	Blinding was not possible in our study. Blinding methods are not established for field studies on biodiversity and not relevant for sampling data of species composition in different plots, since there are no subjective judgements involved that may bias the results across sites.

Did the study involve field work? ☒ Yes ☐ No

Field work, collection and transport

Field conditions	Climatic conditions are typical for moist tropical forests with mean annual temperature of 23°C and mean annual precipitation of 3000–6000 mm.
Location	All 62 plots of the study area are located within a large region of ca. 200 km2. The study is located at 0.5°N 79.2°W, the range of elevation is 130–540 m asl.
Access & import/export	Sample collection, transport and export permits are regulated with a General Contract (Contrato Marco) for all the subprojects with the Ecuadorian Ministry of Environment via the involved institutions (Universidad de las Américas and Pontificia Universidad Católica del Ecuador in Quito). Export permits are Nagoya compatible and are registred via a Due Diligence Declaration in the European Commission (Project ID 158479).
Disturbance	As sampling collections do not target vulnerable species and occur at a small spatial scale, the established survey methods of our study do not represent a significant disturbance to the forest ecosystem nor a threat to regeneration and conservation of this habitat.

Reporting for specific materials, systems and methods

We require information from authors about some types of materials, experimental systems and methods used in many studies. Here, indicate whether each material, system or method listed is relevant to your study. If you are not sure if a list item applies to your research, read the appropriate section before selecting a response.

Materials & experimental systems

n/a	Involved in the study	
☒	☐	Antibodies
☒	☐	Eukaryotic cell lines
☒	☐	Palaeontology and archaeology
☐	☒	Animals and other organisms
☒	☐	Clinical data
☒	☐	Dual use research of concern
☐	☒	Plants

Methods

n/a	Involved in the study	
☒	☐	ChIP-seq
☒	☐	Flow cytometry
☒	☐	MRI-based neuroimaging

Animals and other research organisms

Policy information about studies involving animals; ARRIVE guidelines recommended for reporting animal research, and Sex and Gender in Research

Laboratory animals	No laboratory animals were used in the study
Wild animals	Two of the taxa involved wild vertebrates that were hand collected: bats from mist nets and frogs from the forest floor.<br>We used mist nets to capture bats. They were handled by experts, removed from the nets, kept in clean cloth bags until they could be examined for identification and measurement (about 30 minutes), and then released at the same sampling site.<br>During plot searches by experts (authors of this study), detected frogs were gently pushed into a plastic tube (no direct handling). In order to avoid potential transmission of diseases, each frog was then immediately transferred and temporarily kept in a separate,

clean plastic bag, until plot search was finished. Subsequently, each frog was identified (in the field on the respective plot) to species level, based on external morphology (BioWeb; Ron et al., 2024), sexed (based on species- and sex-specific characters, e.g. vocal sacs, nuptial pads, eggs visible through skin etc.) and measured. To avoid potential transmission of diseases or toxic secretions, we used for each frog a new pair of laboratory gloves. After that procedure the frogs were immediately released on the respective plots. The time from capturing a frog to its release, varied from a few minutes to about an hour. All our work complied with the guidelines for amphibians and reptiles in field research, compiled by the American Society of Ichthyologists and Herpetologists (ASIH), The Herpetologists' League (HL) and the Society for the Study of Amphibians and Reptiles (SSAR) (https://ssarherps.org/wp-content/uploads/2014/07/guidelinesherpsresearch2004.pdf). This established treatment (and collection of specimens) was covered by research and collection permits (MAATE-DBI-CM-2021-0187, add further numbers).
References: Ron, S., A. Merino-Viteri, and A. Ortiz. 2024. "BioWeb, Anfibios Del Ecuador. Versión 2024.0." 2024. https://bioweb.bio/portal/Datos/UsoDatos/.
Invertebrates were sampled by taxon-specific established methods and traps and directly killed and preserved in alcohol or freezer, details of sampling methods are described in the paper. Collection of specimen was covered by research and collection permits.

Reporting on sex

No sex oder gender-specific information has been collected in this study.

Field-collected samples

For taxa that involved collection of samples (invertebrates, bacteria), samples were stored in alcohol or in the freezer in our research station laboratory until they were transfered to Quito or exported to Germany, particularly for DNA analysis and/or for preservation in insect collections hosted by the institutions and museums.

Ethics oversight

Ethical approval or guidance was not required for this study. However, all project participants agreed upon rules of procedure how to handle individual responsibilities and rights such as data ownership and publication ethics, or rules to solve conflicts.

Note that full information on the approval of the study protocol must also be provided in the manuscript.

Dual use research of concern

Policy information about dual use research of concern

Hazards

Could the accidental, deliberate or reckless misuse of agents or technologies generated in the work, or the application of information presented in the manuscript, pose a threat to:

No	Yes	
☒	☐	Public health
☒	☐	National security
☒	☐	Crops and/or livestock
☒	☐	Ecosystems
☒	☐	Any other significant area

Experiments of concern

Does the work involve any of these experiments of concern:

No	Yes	
☒	☐	Demonstrate how to render a vaccine ineffective
☒	☐	Confer resistance to therapeutically useful antibiotics or antiviral agents
☒	☐	Enhance the virulence of a pathogen or render a nonpathogen virulent
☒	☐	Increase transmissibility of a pathogen
☒	☐	Alter the host range of a pathogen
☒	☐	Enable evasion of diagnostic/detection modalities
☒	☐	Enable the weaponization of a biological agent or toxin
☒	☐	Any other potentially harmful combination of experiments and agents

Plants

Seed stocks	N/A
Novel plant genotypes	N/A
Authentication	N/A
